# Mississippi Valley Dental Depot

**Published:** 1857-04

**Authors:** 


*Mississippi Valley Dental Depot.*—We regret to learn, that in consequence of ill health, Dr. A. M. Leslie, formerly a practitioner of Dental Surgery in Cincinnati, Ohio, and well known to most of our readers, as one of the contributors to the journal, has been compelled to relinquish the practice of his profession, but we are gratified to know that he has not entirely disconnected himself from his brethren. It will be seen by an advertisement on the cover of the journal that he has opened a Dental Depot at St. Louis, Mo., under the above designation. We know of no one more competent to conduct an establishment of this kind than Dr. L. His thorough knowledge of the wants of his professional brethren renders him peculiarly qualified for the calling in which he is now engaged, and we take pleasure in directing the attention of our Mississippi Valley readers to his establishment.

